# Web-Based Newborn Screening System for Metabolic Diseases: Machine Learning Versus Clinicians

**DOI:** 10.2196/jmir.2495

**Published:** 2013-05-23

**Authors:** Wei-Hsin Chen, Sheau-Ling Hsieh, Kai-Ping Hsu, Han-Ping Chen, Xing-Yu Su, Yi-Ju Tseng, Yin-Hsiu Chien, Wuh-Liang Hwu, Feipei Lai

**Affiliations:** ^1^National Taiwan UniversityGraduate Institute of Biomedical Electronics and BioinformaticsTaipeiTaiwan; ^2^National Chiao Tung UniversityHsinchuTaiwan; ^3^National Taiwan UniversityComputer and Information Networking CenterTaipeiTaiwan; ^4^National Taiwan UniversityDepartment of Computer Science and Information EngineeringTaipeiTaiwan; ^5^National Taiwan University HospitalDepartment of Medical GeneticsTaipeiTaiwan; ^6^National Taiwan UniversityDepartment of Electrical EngineeringTaipeiTaiwan

**Keywords:** Web-based services, neonatal screening, tandem mass spectrometry, information systems, metabolism, inborn errors

## Abstract

**Background:**

A hospital information system (HIS) that integrates screening data and interpretation of the data is routinely requested by hospitals and parents. However, the accuracy of disease classification may be low because of the disease characteristics and the analytes used for classification.

**Objective:**

The objective of this study is to describe a system that enhanced the neonatal screening system of the Newborn Screening Center at the National Taiwan University Hospital. The system was designed and deployed according to a service-oriented architecture (SOA) framework under the Web services .NET environment. The system consists of sample collection, testing, diagnosis, evaluation, treatment, and follow-up services among collaborating hospitals. To improve the accuracy of newborn screening, machine learning and optimal feature selection mechanisms were investigated for screening newborns for inborn errors of metabolism.

**Methods:**

The framework of the Newborn Screening Hospital Information System (NSHIS) used the embedded Health Level Seven (HL7) standards for data exchanges among heterogeneous platforms integrated by Web services in the C# language. In this study, machine learning classification was used to predict phenylketonuria (PKU), hypermethioninemia, and 3-methylcrotonyl-CoA-carboxylase (3-MCC) deficiency. The classification methods used 347,312 newborn dried blood samples collected at the Center between 2006 and 2011. Of these, 220 newborns had values over the diagnostic cutoffs (positive cases) and 1557 had values that were over the screening cutoffs but did not meet the diagnostic cutoffs (suspected cases). The original 35 analytes and the manifested features were ranked based on *F* score, then combinations of the top 20 ranked features were selected as input features to support vector machine (SVM) classifiers to obtain optimal feature sets. These feature sets were tested using 5-fold cross-validation and optimal models were generated. The datasets collected in year 2011 were used as predicting cases.

**Results:**

The feature selection strategies were implemented and the optimal markers for PKU, hypermethioninemia, and 3-MCC deficiency were obtained. The results of the machine learning approach were compared with the cutoff scheme. The number of the false positive cases were reduced from 21 to 2 for PKU, from 30 to 10 for hypermethioninemia, and 209 to 46 for 3-MCC deficiency.

**Conclusions:**

This SOA Web service–based newborn screening system can accelerate screening procedures effectively and efficiently. An SVM learning methodology for PKU, hypermethioninemia, and 3-MCC deficiency metabolic diseases classification, including optimal feature selection strategies, is presented. By adopting the results of this study, the number of suspected cases could be reduced dramatically.

## Introduction

Newborn screening (NBS) using biochemical markers to detect presymptomatic infants with certain congenital conditions has been performed for almost 50 years. The aim of NBS is to provide early treatment to prevent or ameliorate the long-term consequences of the detected condition [1-3]. Since the pioneering work by Guthrie [4], who discovered that phenylketonuria (PKU) could be detected from dried blood spots collected on filter paper and transported to a testing laboratory, dozens of congenital diseases, including metabolic and infectious diseases, can now be detected in NBS programs [5]. The advent of tandem mass spectrometry (MS/MS) has resulted in a substantial increase in the number of inborn errors of metabolism (IEMs) included in the NBS panel [6], including amino acid disorders, fatty acid oxidation disorders, and organic acid disorders [7].

Although MS/MS can detect many metabolic species and many IEMs, the diseases included in the NBS panels vary from country to country. For example, the American College of Clinical Genetics has proposed that 29 core and 25 secondary conditions be screened [8], whereas only 12 metabolic disorders are included in the German panel [9]. In Taiwan, the Newborn Screening Center of the National Taiwan University Hospital (NTUH) introduced MS/MS-based screening in 2001 [6]. Among the diseases that we can screen, PKU (screened by phenylalanine [Phe] level) and homocystinuria (screened by methionine [Met] level) have been included in the official recommended list [10]. Nevertheless, 3-methylcrotonyl-CoA carboxylase (3-MCC) deficiency, screened by 3-hydroxyisovalerylcarnitine (C5OH) level, has been the most common condition detected in the MS/MS panel [6].

The sensitivity of MS/MS screening for the 20 to 30 diseases included in the American College of Clinical Genetics screening panel varies among the individual diseases [11]. For example, PKU can be detected by the elevation of Phe levels and low or normal tyrosine (Tyr) levels. A timely and highly discriminating method, such as MS/MS, provides better performance than the previously used bacterial inhibitory method [6]. In contrast, Met is a less reliable marker for homocystinuria. Elevation of Met (hypermethioninemia) can occur in various conditions, such as methionine adenosyltransferase deficiency, glycine N-methyltransferase deficiency, S-adenosylhomocysteine hydrolase deficiency, and cystathionine beta-synthase deficiency (classical homocystinuria) [12], and transient elevation sometimes occurs with liver disease. Some conditions may be benign, with no treatment currently recommended. However, there is an estimated 20% false negative rate in homocystinuria neonatal screening [13], and the contributing factors include early discharge from hospital, low protein intake from breast-feeding, and pyridoxine responsiveness. Elevation of C5OH indicates 3-MCC deficiency and several related disorders; in addition, some newborns born to a 3-MCC-deficient mother can also test positive. The majority of patients with 3-MCC deficiency will not develop any signs or symptoms of disease. Therefore, a suitable interpretation and prediction method to decrease false positives and avoid false negatives is crucial for a newborn screening program.

To improve the specificity of screening, there are several approaches. The cutoff scheme has been a popular screening method [14-16]. Two-tier testing and the use of multiple markers can improve sensitivity and specificity [17]. For example, more specific markers, such as total homocysteine, methylmalonic acid, and isovalerylglycine, can be detected in dried blood spots (DBSs) and have reduced the false-positive rate and improved the positive predictive value of NBS for different diseases [18,19]. We also tested the possibility of applying molecular second-tier testing for citrin deficiency and carnitine uptake defects [20]. However, a different testing format is required that is expensive and time consuming. The interpretive tool developed by the Region 4 Genetics Collaborative may help us to enhance the predictive value and minimize the false-positive rate while retaining sensitivity [21]. Machine learning techniques offer another obvious and promising approach for the examination of high dimensional data. Thus, the goal of this paper is to describe feature selection strategies and use support vector machine (SVM) learning techniques to establish the classification models for metabolic disorder screening and diagnoses.

## Methods

The NTUH initiated newborn screening research in 1981 and has performed the nation’s newborn screening of metabolic diseases since July 1985. The NTUH simultaneously holds the responsibilities for national NBS, a phlebotomy hospital, and a referral hospital. The entire screening process workflow and the corresponding functions, relationships, and roles of participants are illustrated in Multimedia Appendix 1. At present, the NTUH Newborn Screening Center provides these services for approximately one-third of the nation’s newborns. Currently, the coverage rate for NBS has improved to 99.9% in the past few years [22], and the NTUH Newborn Screening Center tests more than 70,000 babies every year.

### System Architecture

**Figure 1:**
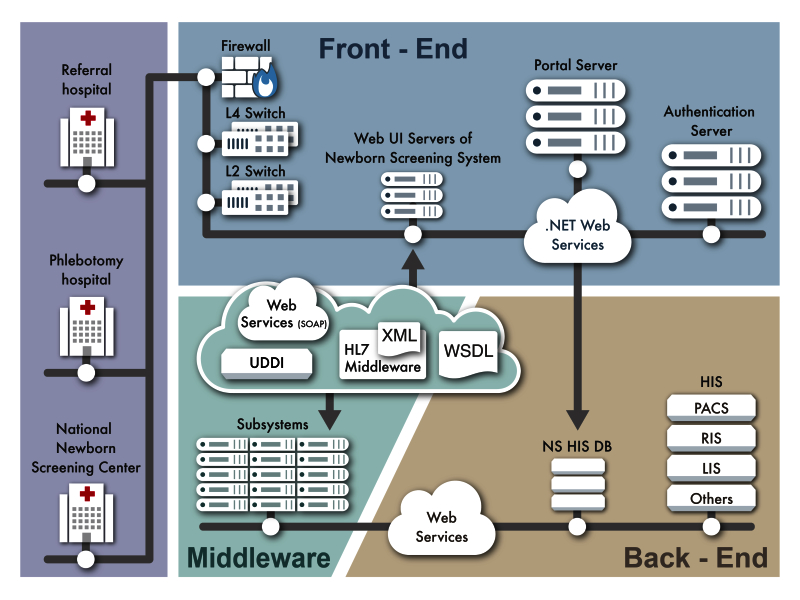
The system architecture of the Web-based newborn screening system.

The overall architecture of the NTUH Newborn Screening Hospital Information System (NSHIS) is depicted in Figure 1. In this diagram, 3 major components, ie, the front-end module, the middleware module, and the back-end services including the database servers are shown. The front-end module handles user interfaces via Web browsers [23] and establishes the users’ sessions with the authentication server. The server validates users’ authentications and authorizations. The middleware module, ie, the Health Level Seven (HL7) middleware framework [24] indicated in the diagram, connects the front-end applications and the back-end facilities. It provides communication and connectivity via a service-oriented architecture (SOA; Web Services) mechanism in the .NET environment. The HL7-embedded Extensible Markup Language (XML)-formatted data are used in the framework for data exchanges among the modules over the simple object access protocol (SOAP) [25,26] and are described in Universal Description, Discovery, and Integration (UDDI) specification with the Web Service Description Language (WSDL). The back-end facilities support services and database storage. The portal server supports the login process with single sign-on service (SSOS) features [27]. The Web user interface server in the architecture generates Web-based pages for users’ interactive activities, including the newborn screening system user interfaces.

In addition, the HL7 middleware framework performs data synchronization between the NSHIS and the NTUH hospital information system (HIS). The NTUH HIS integrates (1) patient demographic data, (2) patient radiology orders in the radiology information system (RIS) database involving the picture archiving and communication system (PACS), and (3) laboratory orders in a laboratory information system (LIS) to ensure data consistency and integrity across the NSHIS and HIS architecture as indicated in the back-end facilities [28-30].

To increase the performance of the NTUH HIS, a cluster of identical servers are deployed and dispatched dynamically by introducing layer 4 (L4) and layer 2 (L2) switches. All the servers are configured to run using load balancing, including failover modes to secure the system’s availability and concurrency. Firewalls are also installed to enhance the security of the architecture.

The NSHIS system is accessible to all authorized screening program professionals and hospitals by enabling the unique identification of babies via screening samples and displaying the results. Authorized users can be doctors, medical staff, administrative personnel, and neonatal parents. The screening hospitals include the Newborn Screening Center, referral hospitals, and phlebotomy clinics as shown on the left in Figure 1. In other words, these members can access the subsystems of NSHIS for services related to their duties, following the newborn screening procedures and after authentication and authorization by the authentication server.

### Data Preparation

The data used in this paper were gathered from the Newborn Screening Center of NTUH between 2006 and 2011 (N=347,312). Dried blood samples from 3-day-old newborns were analyzed by MS/MS in a high-throughput process. The measured metabolic properties (35 measured metabolites including amino acids and acylcarnitines) were archived in the NTUH database. In this study, 3 metabolic diseases (PKU, hypermethioninemia, and 3-MCC deficiency) were reanalyzed. Both screening cutoffs and diagnostic cutoffs were applied in this study. Newborns with an initial screening value that exceeded the diagnostic cutoffs were classified as positive cases and were requested to participate in a confirmation test at our hospital. Newborns with an initial screening value not exceeding the diagnostic cutoff, but equal to or exceeding the screening cutoff, were classified as suspected cases and were asked to undergo another DBS screening. The cutoff values for each disease, the numbers of suspected cases and positive cases observed during this period are summarized in Table 1. During this period, we did not have any reports of false negatives for these 3 diseases.

**Table 1:** Summary of the disease data collected from neonates between 2006 and 2011 (N=347,312).

Disease	Screen markers	Suspected cases	Positive cases	Screening cutoff	Diagnostic cutoff
PKU	Phe	203	38	> 85.02 μM	> 220 μM
Hypermethioninemia	Met	261	40	> 54.12 μM	> 110 μM
3-MCC deficiency	C5OH	1093	142	> 0.56 μM	> 2.2 μM

### Feature Selection Strategies

#### Support Vector Machines

**Figure 2:**
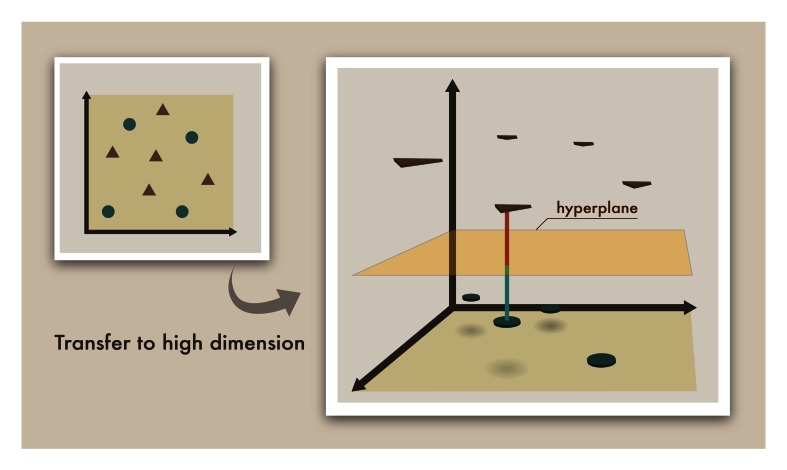
The concept of support vector machine (SVM) methodology is transferring the vectors (ie, cases) to a higher dimension. The optimal linear hyperplane could be obtained from the largest distances between the 2 categories.

An SVM [31,32] performs classification by constructing an N-dimensional hyperplane that optimally separates the data into 2 categories, as shown in Figure 2. In the parlance of the SVM literature, an *attribute* is a predictor variable, and a *feature* is a transformed attribute that is used to define the hyperplane. The task of choosing the most suitable representation is known as *feature selection*. A set of features that describes 1 case (ie, a row of predictor values) is called a *vector*. Therefore, the goal of SVM modeling is to find the optimal hyperplane that separates clusters of vectors. Several different kernel functions can be used, such as linear, polynomial, radial basis function (RBF), and sigmoid. The detailed descriptions of the SVM methodology are presented in Multimedia Appendix 2 [33]. In this study, the machine learning approach only used the SVM RBF kernel. There are 2 input parameters to an SVM: the slack variable (C) is set as default value 100, and the gamma value is set by default to the reciprocal of the number of input features.

#### Data Training and Prediction

**Figure 3:**
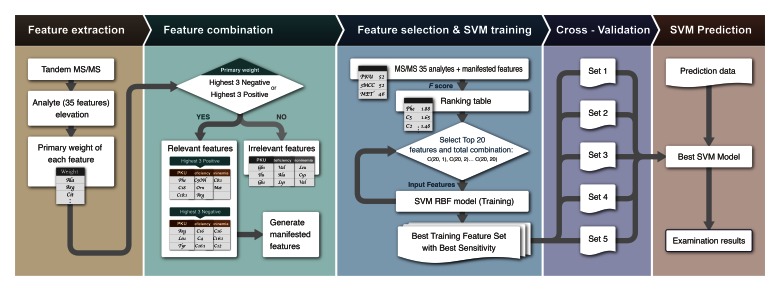
Training and prediction strategies.

In this study, a properly supervised classification dataflow is proposed to enhance the accuracy and sensitivity of the NBS process, as depicted in Figure 3. In the diagram, the training data are used to produce the SVM prediction model. Next, the testing data uses the same methods to obtain the prediction result according to the trained model. Before training or predicting, the dataset is preprocessed by the MS/MS machine and digitization procedures. Feature selection generates the most relevant features.

For classifications, the data are divided into the training, testing, and prediction datasets. In the diagram, the strategies are illustrated step-by-step as follows:

##### Step 1

**Figure 4:**
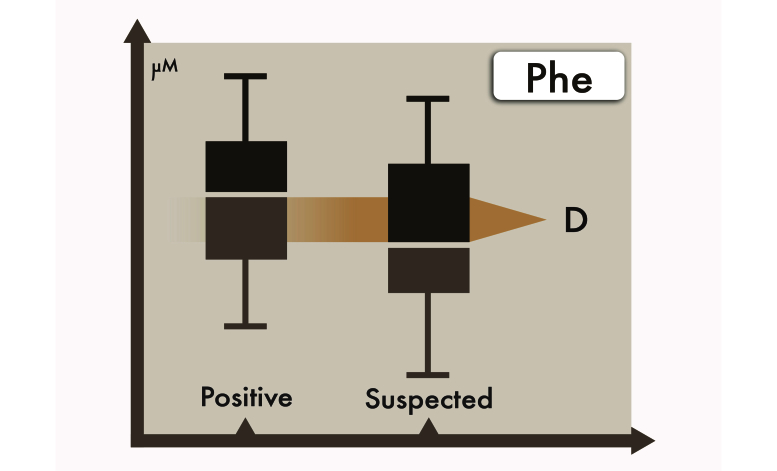
Boxplot of phenylalanine (Phe) showing the difference (D) between positive and suspected cases of the training data.

**Figure 5:**
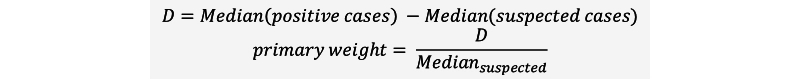
Primary weight formula.

The MS/MS data from positive cases and from suspect cases were used as training (step 3) and testing (step 4) datasets. For each analyte, the difference of the median values between the positive cases and the suspect cases is represented as *D*, as in Figure 4. The primary weight for each analyte was calculated according to the formula defined in Figure 5.

##### Step 2

The highest 3 positive primary weights of the individual diseases among the 35 analytes were (1) PKU: Phe, stearoylcarnitine (C18), and octadecenoylcarnitine (C18:1); (2) 3-MCC deficiency: C5OH, ornithine (Orn), and arginine (Arg); and (3) hypermethioninemia: octenoylcarnitine (C8:1) and Met. In hypermethioninemia, only 2 analytes showed positive primary weights. The highest 3 negative primary weights of the individual diseases among the 35 analytes were (1) PKU: Arg, leucine (Leu), and Tyr; (2) 3-MCC deficiency: C16, C4, and C16:1; and (3) hypermethioninemia: C16, C16:1, and C12.

**Figure 6:**
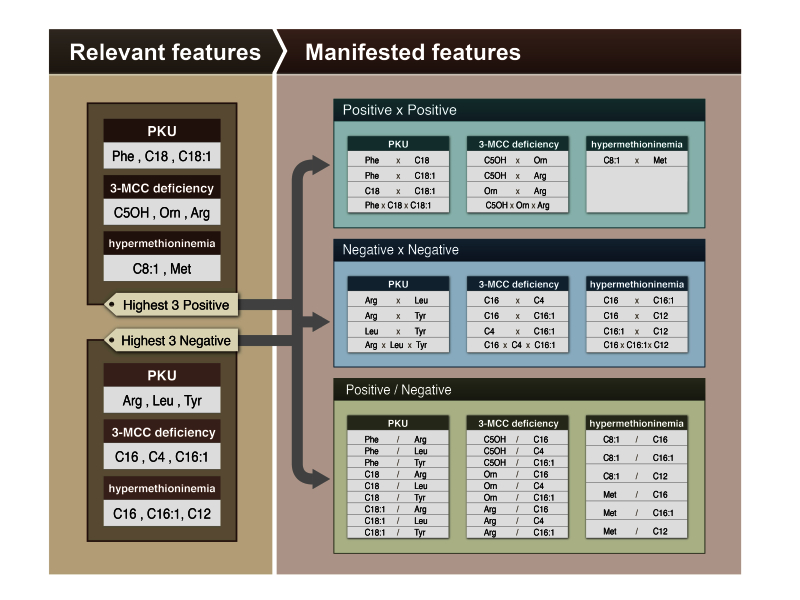
Feature selection strategies by relevant features.

The manifested features are generated by combinations of the highest 3 positives and the highest 3 negatives as listed in Figure 6. The ratios of the highest 3 positives/negatives are also listed in Figure 6. Figure 6 presents the feature selection strategies used to construct the manifested features via the relevant features of the 3 diseases.

##### Step 3

The original 35 analytes and the manifested features were ranked according to the *F* score indicator. The values of the *F* score represent the importance of the features. The top 20 ranked features with the highest *F* scores were selected as the input features. Different combinations of the features, ie, C(20, 1), C(20, 2), C(20, 3),..., C(20, 20), were inputted into the SVM RBF classifier and used to generate the classification model for each combination. Each combination of the features was used as input for the classification models to calculate the sensitivity and specificity. The optimal feature set was defined as the combination with the highest specificity and 100% sensitivity.

##### Step 4

The optimal feature set is then fed into the SVM RBF classifier with 5-fold cross-validation and the optimal model is obtained from the 5 different models of each disease with the highest specificity and 100% sensitivity.

##### Step 5

Based on the optimal model with the optimal feature set, we provided the NBS data collected in 2011 to be used for prediction. The results were classified as either true positive, true negative, false positive, or false negative. The definition of cases was based on the current method and the confirmation results; therefore, we assumed no false negatives were revealed by the current method.

## Results

### Newborn Screening Hospital Information System

A new generation of the NSHIS at the Newborn Screening Center of the NTUH was designed and deployed under a SOA Web services middleware framework. The framework applied embedded HL7 standards in SOAP messages to share data among heterogeneous platforms. The NSHIS successfully provides secure, Web-based, real-time, newborn screening applications in Taiwan.

### Training Results

#### Optimal Feature Sets

According to the described feature selection strategies, the optimal selected markers, ie, optimal feature sets, of the 3 diseases are summarized in Table 2. After following step 4 as described previously, the optimal model for each disease is generated.

### Prediction Results

Of the 347,312 newborn samples collected through the Newborn Screening Center of NTUH from 2006 to 2011, 220 were positive cases and 1557 were suspected cases for the 3 conditions. Because it was a retrospective analysis study, we can only compare the proposed method to the current method to determine if the proposed method has better discriminatory power. After obtaining the optimal model for individual disease from 2006 to 2010, we attempted to predict the disease state for the newborn samples collected in year 2011; the prediction results are listed in Table 3.

In Table 3, the proposed method receives the same sensitivity as that of the cutoff scheme, ie, 100%, for the 3 diseases. Similarly, for specificity and the accuracy, the proposed approach and the cutoff scheme achieve a sensitivity greater than 99% for the 3 diseases in the current experiments.

The effectiveness of the classifier is shown in Table 4. No false positive cases were generated using the proposed approach. Using the proposed approach can significantly decrease the false positive cases for PKU, hypermethioninemia, and especially 3-MCC deficiency.

**Table 2:** Selected markers of the three diseases.

Diseases	Selected markers^a^
PKU	Ala; Met; Phe; C4; C16:1; Leu×Tyr
Hypermethioninemia	Arg; Met; Phe; Val; C4; C8; C10; C10:1; C14; C14:1
3-MCC deficiency	C3; C5OH; C6; C8; C10:1; C14; C14:1; C16×C4; C4×C16:1; Orn/C16

^a^Ala: alanine; Met: methionine; Phe: phenylalanine; Leu: leucine; Tyr: tyrosine; Val: valine; Orn: ornithine; Arg: Arginine;C3: propionylcarnitine; C4: isobutyrylcarnitine; C5OH: 3-hydroxyisovalerylcarnitine; C6: hexanoylcarnitine; C8: octanoylcarnitine; C10: decanoylcarnitine; C10:1: decenoylcarnitine; C14: tetradecanoylcarnitine; C14:1: tetradecenoylcarnitine; C16: palmitoylcarnitine; C16:1: palmitoleylcarnitine.

**Table 3:** Comparison of the current versus the proposed method. The sensitivity, specificity and accuracy are calculated from predicting the neonatal samples of 2011.

Diseases	Methods	Sensitivity (%)	Specificity (%)	Accuracy (%)
PKU	Current	—	99.971	99.971
	Proposed	100	99.997	99.997
Hypermethionemia	Current	—	99.958	99.958
	Proposed	100	99.986	99.986
3-MCC deficiency	Current	—	99.711	99.711
	Proposed	100	99.936	99.936

**Table 4:** Comparison of the current method versus the proposed method. The numbers are obtained from predicting the neonatal samples.

Diseases	Methods	True positives (n)	True negatives (n)	False positives (n)	False negatives (n)
PKU	Current	3	—	21	—
	Proposed	3	72,111	2	0
Hypermethionemia	Current	3	—	30	—
	Proposed	3	72,112	10	0
3-MCC deficiency	Current	6	—	209	—
	Proposed	6	72,255	46	0

## Discussion

### The Newborn Screening Hospital Information System

The NSHIS is a newborn screening information management system providing services and applications to Newborn Screening Center referral hospitals, phlebotomy clinics, and neonatal parents. The system was designed, developed, and deployed based upon middleware, using SOA Web services loosely coupled to technologies in a .NET environment in C# programming language. The HL7 embedded XML formatted data are used in the system for data exchange among the modules over a SOAP request/response mechanism. This system can integrate diverse platforms and databases (eg, NSHIS and the NTUH HIS), and merge, extend, and enhance the accuracy and the reliability of the proposed screening applications. The functionalities include specimen receiving, specimen tracking, uploading of the testing results, screening data management, quality control analyses, classifications, and Web integration. Undoubtedly, the system can provide timely delivery of complete and accurate information for newborn screening. Therefore, the system is able to comprehensively improve the quality of care and well-being of newborns. A scenario for the NSHIS functionalities is presented in Multimedia Appendix 3.

### Proposed Approach

The purpose of this research is to separate apparently healthy individuals who have a disease from those who most likely do not. In addition to the original 35 analytes, we also used different mathematical combinations of the analytes for the manifested features. The use of SVM methods contributes to the credibility of the examination and screening results. During the screening process, the false positive cases will be requested to repeat DBS screening or have a confirmation test. Therefore, these cases will consume additional medical resources and increase parents’ anxiety. By adapting this approach, the number of suspected cases can be reduced substantially; additionally, medical resources will be used effectively and efficiently.

The Mayo Clinic College of Medicine cooperated and collaborated globally to establish a database of unprecedented size containing true positive cases [11]. Based on this database and multivariate pattern-recognition software and through the use of postanalytical and interpretive tools, multiple clinically significant results were compiled into a single score [21]. We also tested these tools and confirmed that they can significantly reduce the false positive cases and eliminate at least half of the cost resulting from unnecessary tests. Although we used only regional data to train our model, after combining our feature selection strategies and the ranked manifested markers, we can estimate the covariance among analytes and decrease the false positive cases. Therefore, we provide another approach to enhance NBS results, although further comparisons using the approach and revisions will be necessary.

### Limitations

The feature selection strategies are initially designed based on mathematical expressions containing the highest 3 positive and negative ranked features to generate the manifested features. The manifested features and the original 35 analytes constitute the total features, for example, PKU has 52 features, whereas hypermethioninemia has 46 features and 3-MCC deficiency has 52 features. Among the total features, the top 20 *F* score–ranked features are selected for the total combinations as input features for training in order to create optimal feature sets for the 3 diseases. Apparently, the feature selection strategies did not include the total features for establishing either the manifested features or total combinations. To consider all of the features, additional human and computational resources are required to enhance the current automated programming methods.

Presently, PKU, hypermethioninemia, and 3-MCC deficiency are the emphasized metabolic diseases. Other diseases were considered; however, several diseases present problems: (1) carnitine transporter defect and citrullinemia cannot be fully detected using blood samples at birth [20], and (2) other diseases, such as medium-chain acyl-CoA dehydrogenase deficiency, cannot be evaluated using the proposed approach because of the low number of training cases.

### Future Work

The NSHIS plans to promote comprehensive care by establishing additional applications for home follow-ups and working with the children with the rare inherited disorders and their families [34]. The exact applications are under evaluation and investigation. Recently, the system has been improved and enhanced to include online billing and charging facilities at phlebotomy clinics and referral hospitals. In addition, the newborn screening test for severe combined immunodeficiency (SCID) has been added to the system. Therefore, the development and deployment of the NTUH NSHIS is evolving as needed.

To assist the research group in exploring further feature selection strategies and machine learning algorithms and in improving classification accuracy, the Computer Center of National Taiwan University offers high-performance computing services. The group members can apply for accounts and use the facilities for both programming and computing resources.

### Conclusion

A new generation of the NSHIS of the Newborn Screening Center at NTUH has been designed and deployed using a SOA middleware framework. The framework applies embedded HL7 standards in SOAP request/response for data exchanges among heterogeneous platforms integrated by Web Services. We have established SVM RBF classifiers using the experimental datasets of 35 original MS/MS analytes and the manifested features. The system supports a set of new methods to refine the screening statistics. The methodology we demonstrated here can effectively enhance screening accuracy and the quality controls without changing the current screening method. The methodology can be easily adapted for routine MS/MS newborn screening. Although we will need other true positive cases to train and define the features for other diseases, we demonstrate the superiority of SVM in this study.

## Multimedia Appendix 1

Newborn screening data workflow process.

## Multimedia Appendix 2

Newborn screening for phenylketonuria: machine learning vs clinicians.

## Multimedia Appendix 3

NSHIS implementation and a scenario.

## Abbreviations

3-MCC deficiency: 3-methylcrotonyl-CoA-carboxylase deficiency
Ala: alanine
Arg: arginine
C5OH: 3-hydroxyisovalerylcarnitine
DBS: dried blood spot
HIS: hospital information system
HL7: Health Level Seven
IEM: inborn error of metabolism
Leu: leucine
LIS: laboratory information system
Met: methionine
MS/MS: tandem mass spectrometry
NBS: newborn screening
NSHIS: Newborn Screening Hospital Information System
NTUH: National Taiwan University Hospital
Orn: ornithine
PACS: picture archiving and communication system
Phe: phenylalanine
PKU: phenylketonuria
RBF: radial basis function
RIS: radiology information system
SCID: severe combined immunodeficiency
SOA: service-oriented architecture
SOAP: simple object access protocol
SSOS: single sign-on service
SVM: support vector machine
Tyr: tyrosine
UDDI: Universal Description, Discovery, and Integration
Val: valine
WSDL: Web Service Description Language
XML: Extensible Markup Language
