# Supplementary material for: Web-Based Newborn Screening System for Metabolic Diseases: Machine Learning Versus Clinicians
Source: J Med Internet Res. 2013 May 23;15(5):e98. doi: 10.2196/jmir.2495 (PMC3668606; doi:10.2196/jmir.2495)
Supplement: Supplementary file 1 [file jmir_v15i5e98_app1.pdf]

Appendix 1- Newborn screening data workflow process

National Taiwan University Hospital (NTUH) simultaneously holds the responsibilities of a National Newborn Screening Center, phlebotomy clinic, and referral hospital; hence its subsystems include all procedures of a newborn screening process, from the very first phlebotomy to the eventual diagnoses. The whole newborn screening process workflow and their corresponding functionalities, relationships as well as roles or members' involvements are illustrated in the following section.

Workflow processes

In **Figure 1**, it elaborates an integrated workflow process of Newborn screening hospital information system of NTUH. The process can be categorized into several stages; the functionalities of each stage are included in the diagram. The involving roles, their relationships, and activities are indicated as well.

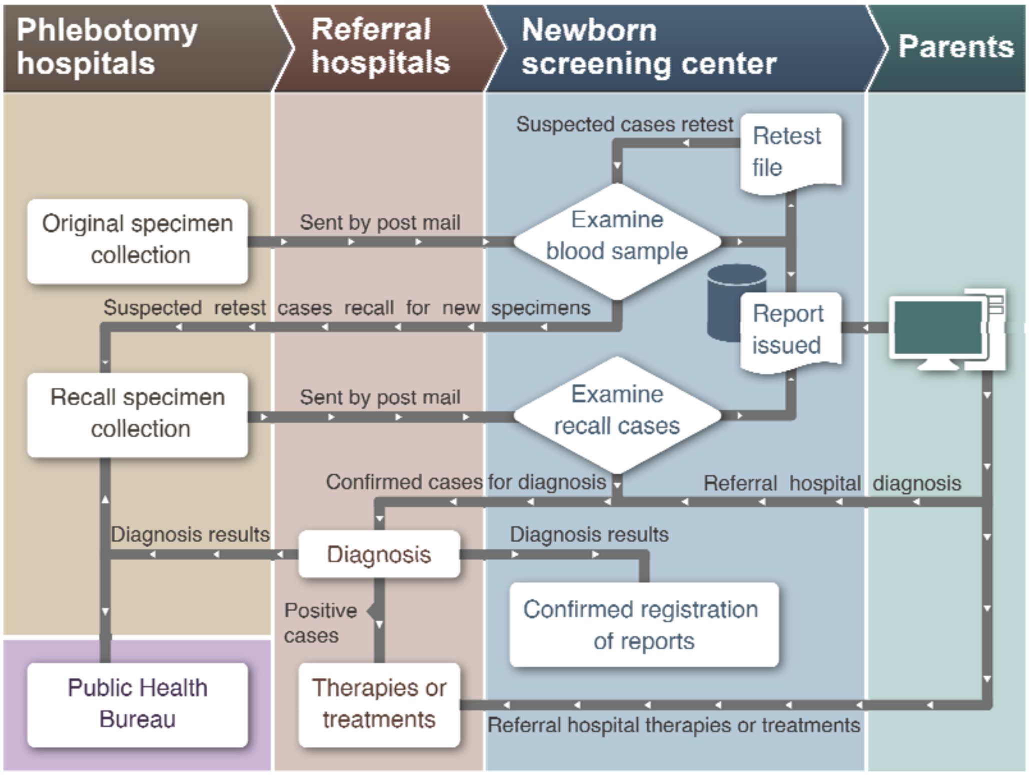

Figure 1. Workflow of newborn screening processes in NTUH

1) Specimen Collection: initially, the phlebotomy hospitals collect specimens, and fill out infants' demographic data (i.e., his/her mother's name, date of birth, weight, etc.) in the phlebotomy filter paper. Afterwards, the hospitals deliver the newborns'

phlebotomy filter papers to the National Newborn Screening Center at NTUH via the post mail service daily. As soon as the center receives the specimens, laboratory serial numbers are assigned to each baby's specimen. Meanwhile, the newborns' information is entered into the NTUH database manually.

2) Sample Screening: secondly, technicians inject the specimens into the experimental apparatus, i.e., MS/MS (tandem mass spectroscopy); the system can accommodate 96-well microtiter plates. After analyzing the blood compound, the system generates the raw data tabulated into a single spreadsheet of concentration. The data are examined and validated via statistical methods applied to establish the upper limits for each analyte (also referred to as the "cutoffs"). If the outcome is negative, the case is directly stored into the database and issued a report; parents and doctors can review the status online. This completes the newborn screening procedures.

3) Suspected Case Retest: If the outcome is beyond the cutoff target ranges, concerned instrumental misjudgments or errors, the specimen will undergo a second examination; a Retest file is generated. The case will be re-examined on the following day. In addition, during the process, a list of reports is recorded. The reports assist doctors and technicians to clinically validate the cases later. All the Retest values will replace the previous ones.

4) Test Result Recalled: based on the primary test results, the suspected positive cases are interrogated and verified by laboratorians. The cases return to the original phlebotomy hospitals. The phlebotomy hospitals are responsible for tracking and obtaining a new sample as Recalled specimens. The specimens will be delivered to the center for additional examinations. If the results turn out to be positive or suspected again, the case will be forwarded to the doctors for further diagnoses. The parents of the newborns and the associated hospital can track the results, a report issued, online within three days.

5) Diagnosis Confirmation: for positive cases, a referral hospital is recommended and is in charge of follow-up the cases. The hospital provides preliminary precaution advices and comprehensive confirmation diagnoses for the cases. The confirmation reports will be issued at the National Newborn Screening Center, Public Health Bureau, and the original phlebotomy hospitals via e-mail. The case will be registered at the center as well. Moreover, the patients will receive further dietary therapies or treatments. Thus, it completes the whole newborn screening procedures.
