# Supplementary material for: Web-Based Newborn Screening System for Metabolic Diseases: Machine Learning Versus Clinicians
Source: J Med Internet Res. 2013 May 23;15(5):e98. doi: 10.2196/jmir.2495 (PMC3668606; doi:10.2196/jmir.2495)
Supplement: Supplementary file 2 [file jmir_v15i5e98_app2.pdf]

Newborn Screening for Phenylketonuria: Machine Learning vs Clinicians

[1]Wei-Hsin Chen, [2]Han-Ping Chen, [1]Yi-Ju Tseng, [3]Kai-Ping Hsu, [5]Sheau-Ling Hsieh, [6]Yin-Hsiu Chien, [6]Wuh-Liang Hwu, [1,2,4]Feipei Lai

[1]Graduate Institute of Biomedical Electronics and Bioinformatics,
[2]Department of Computer Science and Information Engineering,
[3]Computer and Information Networking Center
[4]Department of Electrical Engineering
National Taiwan University, Taipei, Taiwan
[5]National Chaio Tung University, Hsinchu, Taiwan
[6]Department of Medical Genetics, National Taiwan University Hospital, Taipei, Taiwan
paranois@gmail.com

***Abstract*—The metabolic disorders may hinder an infant's normal physical or mental development during the neonatal period. The metabolic diseases can be treated by effective therapies if the diseases are discovered in the early stages. Therefore, newborn screening program is essential to prevent neonatal from these damages. In the paper, a support vector machine (SVM) based algorithm is introduced in place of cut-off value decision to evaluate the analyte elevation raw data associated with Phenylketonuria. The data were obtained from tandem mass spectrometry (MS/MS) for newborns. In addition, a combined feature selection mechanism is proposed to compare with the cut-off scheme. By adapting the mechanism, the number of suspected cases is reduced substantially; it also handles the medical resources effectively and efficiently.**

Keywords- Newborn screening, Tandem mass spectrometry, Support Vector Machine

I. INTRODUCTION

Newborn screening programs for severe metabolic disorders, that hinder an infant's normal physical or mental development, are well established [1-3]. The metabolic diseases can be treated by effective therapies at the early stages. For most patients, if not being diagnosed and treated in time, the acute encephalopathy crises can occur during infancy or early childhood.

New and refined screening methodologies based on the modern tandem mass spectrometry (MS/MS) of metabolites have been developed for routine deployment [4-7]. It allows simultaneous analyses of multi-compounds in a high-throughput process. The functional endpoint of the metabolic cycles, offering a precise snapshot of the current metabolic state, can be detected, analyzed in a single, small blood sample. The sample is collected during the first few days of life. Over the last two decades, this technology has been revolutionary and has resulted in remarkable expansion to detect metabolic disorders [7-8]. It is open to population-wide testing for a large number of disorders [9].

The National Taiwan University Hospital (NTUH) initiated the newborn screening research in 1981. It has carried on the nation's newborn screening tasks for metabolic diseases since July, 1985. At present, NTUH newborn screening laboratory provides medical service to approximately one third of the nation's newborns for inborn errors of metabolism. Moreover, in Taiwan, the screening rate of newborn's inherited metabolic diseases has improved from 6.4% in 1984 to reach over 99.9% in the past years. Currently, the screening program executed in Taiwan, all newborns are tested for inherited metabolic disorders [10]. Each year the NTUH Newborn Screening Program tests over 70,000 babies and about 30-40 babies need urgent assessments and treatments.

Phenylketonuria (PKU) detection is universally included in the newborn screening items over majority countries applying tandem mass spectrometry. The analyte elevations of Phenylalanine (PHE) and the ratio of PHE to Tyrosine (TYR) determine the PKU. In general, a newborn has higher values of both analyte elevations; the baby has stronger tendency of having the PKU disease. If the baby is not screened during the routine newborn screening examination, the disease may occur clinically with seizures, albinism (excessively fair hair and skin), and a "musty odor" of the baby's perspiration and urine. In most cases, a follow-up examination should be done at approximately two weeks of age to verify the initial result and uncover any PKU that was initially missed. Untreated children are normal at birth, but fail to attain early developmental milestones, develop microcephaly, and demonstrate progressive impairment of cerebral function. Hyperactivity, EEG abnormalities and seizures, and severe learning disabilities are major clinical problems later in the children's lives.

Machine learning techniques offer an obvious and promising approach to examine high dimensional data. Thus, the goal of the paper is to utilize machine learning techniques and mining knowledge to establish the classification models for metabolic disorders screenings and diagnoses. The models can

generate high discrimination and improve prediction accuracies. The support machine machines (SVM) is a robust technique for data classification. An important concept for SVM is to transform the input space into a higher dimensional feature space to realize the linearity of the classifier. SVM will determine an optimal hyperplane for separating binary classes by inputting a training data set. Although excellent results have been achieved based on this classifier for solving these problems in several areas, very few published papers use SVM as the classifier for the newborn screening data.

The remainder of the article is organized as follows. The related work on global newborn screening and cut-off scheme are presented in Section 2. In Section 3, it elaborates data and methodology of the study. Detailed descriptions of the approaches including data collection, digitizing process as well as data training and prediction are illustrated in the section. In Section 4, experimental results are summarized. Finally, the article concludes in Section 5.

II. Related Work

A. Newborn Screening Program

The newborn screening program represents one of the major advances in child health of the past century. The program is a system that must function within geographic, economic, and political constraints, and which must smoothly integrate sample collections, laboratory analyses, follow-up diagnoses and treatments [4, 11].

In the United States (US), the newborn screening program has been carried out in all fifty states since the 1970s [4, 11-13]. However, the programs are state-run, and decisions are left to the individual states regarding the conditions to be screened for, the mechanism for confirmatory testing, and financing of the programs [13-15]. For instance, a total of 31 states required testing for more than 20 of disorders as of June 1, 2006 [16]. The Georgia Newborn Screening Program has educational and monitoring mechanisms in place to remain watchful for any signs or symptoms of disorders in their patients [17]. The March of Dimes recommends that all newborns be screened for 29 disorders including hearing loss. Furthermore, it is estimated that one baby in 10,000 to 25,000 is born with PKU in the US; newborn screening for PKU is required in all 50 states and US territories [1]. In conclusion, each state in the US requires screening tests, but the specific tests performed vary among the states.

The newborn screening program in Asia Pacific, most countries are approaching full coverage, although the number of conditions screened varies widely. However, in the developing countries, Nepal and India are particularly interested in developing and inquiring Congenital Hypothyroidism (CHT) programs. CHT is the universal important condition; usually it is the first condition considered for screening [1, 18].

In Europe, the number of disorders screened for by MS/MS ranges from two disorders (PKU and MCADD (medium-chain acyl-CoA dehydrogenase deficiency)) in some countries to 20 in others [3]. Thus, the disorders chosen to be included in European newborn screening programs differ considerably. For example, PKU and MCADD are examined in Switzerland.

B. Cut-off Scheme

The cut-offs scheme has been a popular screening decision [19-21]. However, manipulating of cut-offs is only one of several strategies to improve specificity of screening without sacrificing sensitivity. Two-tier testing and use of multiple markers improves sensitivity and specificity [22], [23]. For examples, steroid profiling using MS/MS or high performance liquid chromatography (HPLC) following elevated 17-hydroxyprogesterone (17-OHP) in screening for congenital adrenal hyperplasia (CAH) has been shown to be effective. However, the use of the second-tier testing must be done in conjunction with clinical observation of the cases [24]. A three-tiered cut-offs scheme was used during the initial 24 months of CAH screening in Bavaria [25]. In general, while setting cutoffs, a balance must be struck between time, money, anxiety caused by false positives, and an acceptable number of missed cases [17].

III. Data and Methodology

A. Data Collection

In Fig. 1, it illustrates the newborn screening data collection workflow process of NTUH. The process can be categorized into several stages; the functionalities of each stage are included in the diagram.

1) Specimen Collection

Initially, the phlebotomy hospitals collect specimens, and fill out infants' demographic data (i.e., his/her mother's name, date of birth, weight, etc.) in the phlebotomy filter paper. Afterwards, the hospitals deliver the newborns' phlebotomy filter papers to the National Newborn Screening Center at NTUH via the post mail service daily. As soon as the center receives the specimens, laboratory serial numbers are assigned to each baby's specimen. Meanwhile, the newborns' information is entered into the NTUH database manually.

2) Sample Screening

Secondly, technicians inject the specimens into the

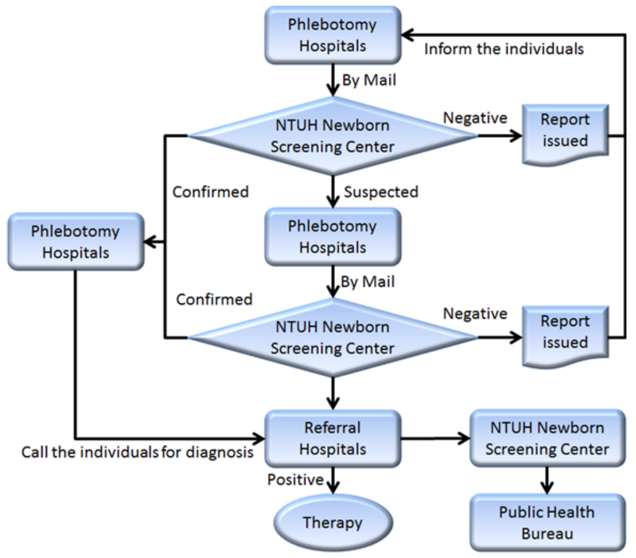

Figure 1. Newborn screening data collection workflow

experimental apparatus, i.e., MS/MS. After analyzing the blood compound, the system generates the raw data of the analyte concentration. The data are examined and validated via statistical methods applied to establish the upper limits for each analyte (also referred to as the "cutoffs"). If the outcome is negative, the case is directly stored into the database and issued a report; parents and doctors can review the status online. This completes the newborn screening procedures.

3) Test Result Recalled

Based on the primary test results, the suspected positive cases are interrogated and verified by laboratorians. The cases return to the original phlebotomy hospitals. The phlebotomy hospitals are responsible for tracking and obtaining a new sample as Recalled specimens. The specimens will be delivered to the center for additional examinations. If the results turn out to be positive or suspected again, the case will be forwarded to the doctors for further diagnoses. The parents of the newborns and the associated hospital can track the results, a report issued, online within three days.

4) Diagnosis Confirmation

For positive cases, a referral hospital is recommended and is in charge of follow-up the cases. The hospital provides preliminary precaution advices and comprehensive confirmation diagnoses for the cases. The confirmation reports will be issued at the National Newborn Screening Center, Public Health Bureau, and the original phlebotomy hospitals via e-mail. The case will be registered at the center as well. Moreover, the patients will receive further dietary therapies or treatments. Thus, it completes the whole newborn screening procedures.

B. Digitization Process

In NTUH, between May 2002 and July 2007, the neonatal screening data are archived both on papers and in CD disks as Microsoft Excel Files (.xls). During the period, technicians manually enter serial numbers of blood spot specimens of 96-well microtiter plate in a report. Before data analysis, these data should be digitized in advance. The digitization process is shown in Fig. 2.

Initially, technicians manually enter serial numbers of blood spot specimens of 96-well microtiter plate on a report. Each serial number identifies an individual baby's specimen. The report consists of 12 wells per row, 8 rows per report, and 35 species per specimen. After MS/MS analyzing, the system generates a single spreadsheet displaying the concentrations of blood compound in a tabulated data format: 1 row per page, total 8 pages per plate as indicated in Fig. 2. The status report of the MS/MS results, i.e., serial numbers, report dates, and the result outcomes, is stored into the NTUH database.

C. Data Training and Prediction

In the study, a proper supervised classification data flow is proposed to enhance the accuracy and sensitivity of newborn screening process, as depicted in Fig. 3. In the diagram, the training data undergoes learning to produce the SVM prediction model; the testing data processes the same methods to obtain the prediction result according to the trained model. Before training or predicting, the dataset is preprocessed by the MS/MS machine and digitization procedure. The feature selection generates the most relevant features. The scaling method is applied to avoid biasing and to improve computing efficiency.

1) Feature Selection

An equation defined in (1) is proposed to evaluate the importance $I(a,b,k)$ [26] of a specific feature k, $1 \leq k \leq m$ for a hyperplane between corresponding class a and class b, where $u_{a,k}$ denotes the mean value of the feature k for all training samples in class a and $v_{a,k}$ is the variance value of the feature k for all the training samples in class a.

$$I(a,b,k) = \frac{(u_{a,k} - u_{b,k})^2}{v_{a,k} + v_{b,k}} \tag{1}$$

This equation aims to evaluate the differentiation capability between the two classes as well as the stability in the same class for a given feature k. For each hyperplane, the

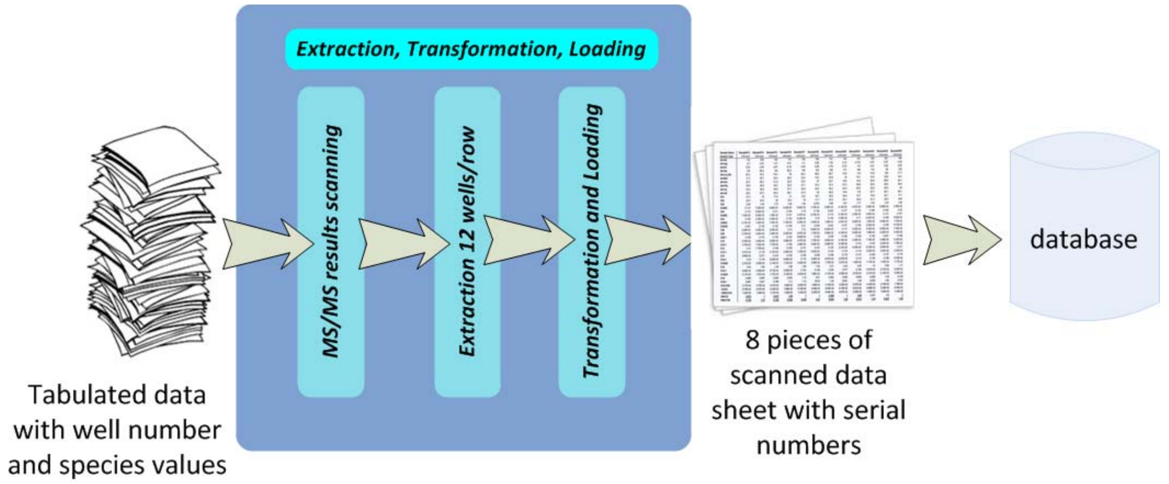

Figure 2. Data digitization procedure.

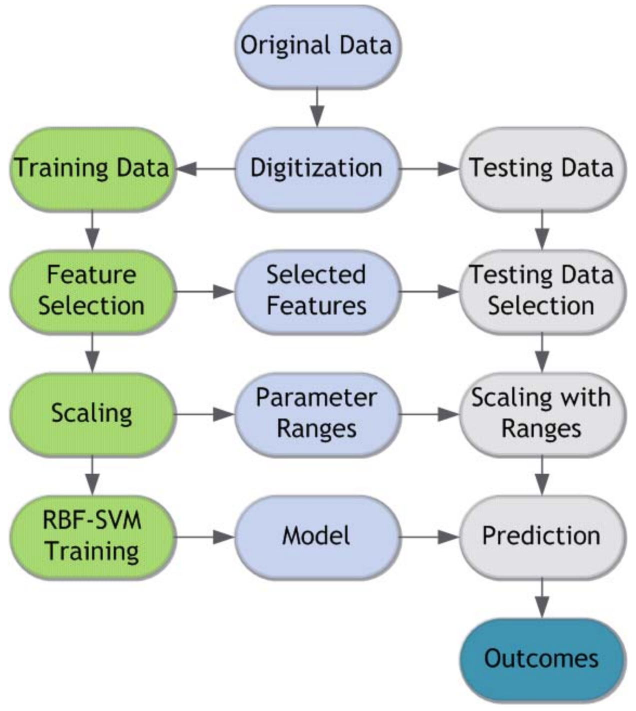

Figure 3. Data Training and Prediction workflow.

importance of each feature is needed to be calculated first, and then they are being sorted by descending order.

2) Scaling

The scale class contains *y_lower*, *y_upper*, *y_min* and *y_max* to store scale range to lower and upper bound and minimum, maximum value of label *y*, respectively. It scales the range from [min, max] to [lower, upper]. The *v* is the original value; *v'* is the value after scaling.

The array *feature_max* and *feature_min* store the maximum and minimum values of the concentration values of each metabolism. The default scale is the concentration value of each metabolism to [lower, upper] ([-1, +1]). The scale class constructor *svm_scaling*() receives dataset from SVM training and SVM predicting and returns the scaled dataset back.

3) Support Vector Machines

The central idea of SVM classification is to use a linear separating hyperplane to create a classifier. The vector which can affect the separation is called a "support vector".

Given a training set of instance-label pairs:

$$(x_i, y_i), i = 1, \dots, l \quad x_i \epsilon R^n, y \epsilon \{1, -1\}^l \tag{2}$$

The hyperplane can be expressed as below:

$$< w^T \cdot x > +b = 0, \tag{3}$$

where $w = [w_1, w_2, \dots w_n]^T, x = [x_1, x_2, \dots x_n]^T$.

Then the definition of a decision function is:

$$f(\mathbf{x}) = sign(w^T q(x) + b) \tag{4}$$

with the largest possible margin, which apart from being an intuitive idea has been shown to provide theoretical guarantees in terms of generalization ability.

If the data are distributed in a highly nonlinear way, employing only a linear function causes many training instances to be on the wrong side of the hyperplane, which results in under-fitting occurs; the decision function does not perform well. Thus, SVM non-linearly transforms the original input into a higher dimensional feature space. More precisely, the training data x is mapped into a (possibly infinite) vector $Q(x) = (q_1(x), q_2(x), \dots, q_i(x), \dots)$.

In this higher dimensional space, it is possible that data can be linearly separated. Therefore, it tries to find a linear separating plane in a higher dimensional space.

$$\begin{gathered} \min_{w,b,s} \frac{1}{2} w^T w + C \sum_{i=1}^{l} s_i \\ \text{subject to } y_i(w^T x_i + b) \geq 1 - s_i, s_i \geq 0 \end{gathered} \tag{5}$$

The parameters s_i are called the slack variables, and they ensure that the problem has a solution in case the data are not linearly separable. The constraints in (5) contain a penalty term, $C \sum_{i=1}^{l} s_i$, where $C > 0$, and the parameter is chosen by the user to assign a penalty to errors. Usually this problem is called a primal problem. After the data are mapped into a higher dimensional space, the number of variables (w, b) becomes very large or even infinite. This difficult is handled by solving the dual problem.

A kernel for a nonlinear SVM projects the samples to a feature space of higher dimension via a nonlinear mapping function. Among the nonlinear kernels, the radial-based function (RBF) is defined as

$$K(x_i, x_j) = \exp\left(-\mathrm{r}\|x_i - x_j\|^2\right), \mathrm{r} > 0, \tag{5}$$

where r is a kernel parameter.

4) SVM Prediction

The final prediction equation for a hyperplane between two classes is expressed by (6), where z is an unknown input vector, x_i are support vectors which m and n denote the number of support vector for the corresponding two classes of the hyperplane, and the kernel function $K(x_i, z)$ is used by RBF in the study.

$$y = \sum_{i=1}^{n+m} A_i y_i K(x_i, z) + b \tag{6}$$

IV. EXPERIMANTAL RESULTS

A. Data sets

The experimental dataset was gathered from the Newborn Screening Center of National Taiwan University Hospital between 2006 and 2010. The blood samples, taken within a few days after the newborns' birth, have been analyzed by MS/MS in a high throughput process. The measured metabolic datasets (35 measured metabolites including amino acids and acylcarnitines) have been archived in the NTUH database. In

this study, the metabolic disease: Phenylketonuria (PKU) is emphasized.

B. Feature Selection Procedure

Before SVM training, Fisher Score [26] is applied for evaluating the importance of the features. The importance scores between the positive and negative cases are illustrated in Table I. PHE is the most significant analyte ranked by Fisher Score and also in original NTUH newborn screening cut-off scheme.

After calculating the importance of the features, several examinations are applied to obtain the best combination feature set in order to raise the accuracy and sensitivity of the training model. Finally, the best combination is LEU, PHE, TYR, C5, C14OH, and C18:1OH.

C. Results

During the experiment, the Phenylketonuria (PKU) metabolic disorder is specified preliminarily. There are 275,205 newborn samples collected from Newborn Screening Center of National Taiwan University Hospital between 2006 and 2010. The samples are divided into two parts, half for training and the other half for prediction. The discriminatory power of the investigated techniques is evaluated by constructing a classification table for the two class problem: true positives (TPs), true negatives (TNs), false positives (FPs) and false negatives (FNs). The most frequently used evaluation measure in classification is accuracy (ACC): ACC = (TP + TN) / (TP + FP + TN + FN). Measures of sensitivity (SEN) have been considered primarily by clinicians. In other words, clinicians insist on detecting of the accuracy of the positive cases, i.e., measure of sensitivity: SEN = TP / (TP + FN). Specificity: SPE = TN / (TN + FP) is the fraction of actual negative samples that are correctly classified. The effectiveness of the classifier is shown in Table II.

TABLE I. FISHER SCORE RANKING

Species	*Scores*	*Species*	*Scores*
PHE	1.8753	C05	1.6566
C02	1.4894	TYR	1.2307
C14OH	1.0565	LEU	0.9307
C10	0.9204	C12:1	0.8546
C05DC	0.8324	C16	0.8193
MET	0.8103	VAL	0.8051
C16:1	0.7213	C01	0.5939
C16OH	0.5938	C18	0.5819
C18:1OH	0.5324	CIT	0.5024
ALA	0.3971	C10:1	0.3947
ARG	0.3461	C04	0.3412
C14:1	0.2821	C08	0.2794
C03DC	0.2350	C18:1	0.1679
GLY	0.1390	C04DC	0.1345
C06	0.1032	ORN	0.0993
C14	0.0757	C08:1	0.0573
C05OH	0.0528	C12	0.0509
C03	0.0346		

In Table II, the first row presents the cases that adapt the traditional cut-off scheme in hospital; the second row is the SVM classification without feature selection; the last row means the results of classifying applying SVM with feature selection. The sensitivity column indicates that the SVM with feature selection method receives the same sensitivity with cut-off scheme, i.e., 100%. In the specificity column displays that the SVM approach with feature selection achieves 99.98% compared with the classical cut-off technique as listed. Similarly, in the table, the SVM without feature selection performs the highest accuracy. However, the approach obtained the sensitivity 82.35%. It implies the method cannot detect positive cases precisely. Obviously, the SVM approach with higher dimensional selected features demonstrates the best discrimination power among the three methodologies. In addition, it increases the accuracy accordingly. The detailed numbers of TN, TP, FN, and FP are demonstrated in Table III.

In the table, the numbers of the FN cases under the cut-off scheme and SVM with feature selection method are 0. It implies the both approaches can predict positive cases accurately. However, the SVM without feature selection method detects 14 positive cases and 3 false negative cases. In other words, the method cannot precisely predict the positive cases. Consequently, it is not acceptable for clinicians.

Furthermore, in the table, the number of FP cases using the cut-off scheme is 100; the same number of the SVM with feature selection method is 35. It indicates that there are 65 more cases have been diagnosed as positive under the cut-off scheme. During the screening process, these cases will be performed follow-up. Subsequently, it generates additional medical resources consumption.

The purpose of the study is to sort out apparently healthy individuals who do not have PKU from those who probably do have. However, screening programs are, by nature, imperfect. In setting cutoffs, a balance must be struck between time, money, anxiety caused by false positives, and an acceptable number of missed cases. On one hand, laboratory advances in tandem mass spectrometry make it possible to screen newborns for many rate inborn errors of metabolism. This raises many policy issues including screening's cost-effectiveness, ethics, quality, and oversight. On the other hand, new techniques in genetics surveillance have facilitated an improved public health

TABLE II. EXPERIMENTAL RESULTS

Methods	*ACC (%)*	*SEN (%)*	*SPC (%)*
PHE (cut-off)	99.96	100	99.96
SVM without Feature Selection	99.99	82.35	99.99
SVM with Feature Selection	99.98	100	99.98

TABLE III. EXPERIMENTAL RESULTS

Methods	*TN*	*TP*	*FN*	*FP*
PHE (cut-off)	275,070	17	0	100
SVM without Feature Selection	275,159	14	3	11
SVM with Feature Selection	275,135	17	0	35

approach to the detection of, and interventions offered for, a range of important genetic conditions. Over the past two decades, scientific advances associated with genetic have been increasing at an explosive rate. It means that an increasing number of diagnostic, predictive and carrier tests are available, for instance, leaning towards data mining technologies.

V. CONCLUSION

In the study, a newborn screening approach is proposed. The approach can predict whether the newborn has metabolic disorder diseases based on Support Vector Machines (SVM) classifier with Fisher Score ranked feature selection. Applying the approach, the predicting accuracy of PKU can be improved from 99.96% (cut-off value approach) to over 99.98%, the number of the false positive cases can be reduced from 100 to 35. Therefore, by adapting the approach, the number of suspected cases is reduced substantially; it also handles the medical resources effectively and efficiently.

ACKNOWLEDGEMENT

The authors would like to acknowledge the partial funding supported from the Microsoft Research Asia and their staff assistances. In addition, the authors would also thank Prof. Chih-Jen Lin and his research team members for kindly providing the LIBSVM tool.

REFERENCES

[1] Kids Health for Nemours Newborn Screening Tests. Available: http://www.kidshealth.org/parent/system/medical/newborn_screening_tests.html.

[2] The NSW Newborn Screening Programme. Available from: http://www.chw.edu.au/prof/services/newborn/.

[3] O. A. Bodamer, G. F. Hoffmann, M. Lindner, "Expanded newborn screening in Europe 2007," Journal of Inherited Metabolic Disease, vol. 30, no. 4, pp. 439-444, 2007.

[4] Expanded Newborn Screening using Tandem Mass Spectrometry Financial, Ethical, Legal and Social Issues (FELSI). Available: http://www.newbornscreening.info.

[5] C. -M. Tu, "The New Generation of Information System for Newborn Screening - A Case Study of National Taiwan University Hospital," M.S. thesis, Dept. Computer Science and Information Engineering, National Taiwan University, Taiwan, 2007.

[6] D. S. Millington, R. D. Stevens, "Acylcarnitines: Analysis in Plasma and Whole Blood Using Tandem Mass Spectrometry," Methods in Molecular Biology, vol. 708, pp.55-72, 2011.

[7] D. S. Millington, "Rapid and Effective Screening for Lysosomal Storage Disease: How Close Are We?," Clinical Chemistry, vol. 54, no. 10, pp. 1592-1594, 2008.

[8] L. Sweetman, D. S. Millington, B. L. Therrell, W. H. Hannon, B. Popovich, M. S. Watson, et al., "Naming and counting disorders (conditions) included in newborn screening panels," Pediatrics, vol. 117, no. S3, pp. S308-S314, 2006.

[9] D. S. Millington, "Tandem Mass Spectrometry in Clinical Diagnosis," in: Physicians Guide to the Laboratory Diagnosis of Metabolic Diseases, 2nd ed. Berlin, Springer, 2003, pp. 57-75.

[10] National Taiwan University Hospital Newborn Screening Center. Available: http://www.ntuh.gov.tw/gene/nbsc/screen/item.html.

[11] J. J. Stoddard, P. M. Farrell, "State-to-state variations in newborn screening policies," Archives of Pediatrics and Adolescent Medicine, vol. 151, no. 6, pp.561-564, 1997.

[12] U. S. Birth Rates and Population Growth. Available: http://www.susps.org/overview/birthrates.html.

[13] P. H. Arn, "Newborn Screening: Current Status," Health Affairs, vol. 26, no. 2, pp. 559-566, 2007.

[14] Medline Plus Newborn Screening Tests. Available: http://www.nlm.nih.gov/medlineplus/ency/article/007257.htm.

[15] DrGreene.com Newborn Screening Tests. Available: http://www.drgreene.com/qa/newborn-screening-tests.

[16] States Doubled Number of Newborns Tested for Genetic Diseases. Available: http://www.foxnews.com/story/0,2933,202900,00.html.

[17] State of Georgia Newborn Screening Program. Available: http://health.state.ga.us/programs/nsmscd/.

[18] Genetics Services in Victoria. Available from: http://www.health.vic.gov.au/genetics/strategy.htm.

[19] S. Ohkubo, K. Shimozawa, M. Matsumoto, T. Kitagawa, "Analysis of blood spot 17α-hydroxyprogesterone concentration in premature infants -proposal for cut-off limits in screening for congenital adrenal hyperplasia," Acta Paediatrica Japonica, vol. 34, pp. 126-133, 1992.

[20] J. A. Lott, M. Sardovia-Iyer, K. S. Speakman, K. K. Lee, "Age-dependent cutoff values in screening newborns for Hypothyroidism," Clinical Biochemistry, vol. 37, no. 9, pp. 791-797, 2004.

[21] M. Lindner, S. Ho, J. Fang-Hoffmann, G. F. Hoffmann, S. Kolker, "Neonatal screen for glutaric aciduria type I: Strategies to proceed," Journal of Inherited Metabolic Disease, vol. 29, no. 2-3, pp. 378-382, 2006.

[22] S. A. McGhee, E. R. Stiehm, M. Cowan, P. Krogstad, E. R. B. McCabe, "Two-tiered universal newborn screening strategy for severe combined immunodeficiency," Molecular Genetics and Metabolism, vol. 86, no. 4, pp. 427-430, 2005.

[23] G. Marquardt, R. Currier, D. M. S. McHugh, D. Gavrilov, M. J. Magera, D. Matern, et al., "Enhanced interpretation of newborn screening results without analyte cutoff values," Genetics in Medicine, accepted 2012.

[24] D. Webster, "Quality performance of newborn screening systems: Strategies for improvement," Journal of Inherited Metabolic Disease, vol. 30, no. 4, pp.576-584, 2007.

[25] B. Olgemoller, A. A. Roscher, B. Liebl, R. Fingerhut, "Screening for Congenital Adrenal Hyperplasia: Adjustment of 17-Hydroxyprogesterone Cut-Off Values to Both Age and Birth Weight Markedly Improves the Predictive Value," Journal of Clinical Endocrinology and Metabolism, vol. 88, no. 12, pp. 5790-5794, 2003.

[26] P. E. H. R. O. Duda and D. G. Stork. Pattern Classification. Wiley-Interscience Publication, 2001.
