# Supplementary material for: Web-Based Newborn Screening System for Metabolic Diseases: Machine Learning Versus Clinicians
Source: J Med Internet Res. 2013 May 23;15(5):e98. doi: 10.2196/jmir.2495 (PMC3668606; doi:10.2196/jmir.2495)
Supplement: Supplementary file 3 [file jmir_v15i5e98_app3.pdf]

Appendix 4 - NSHIS implementation and a Scenario

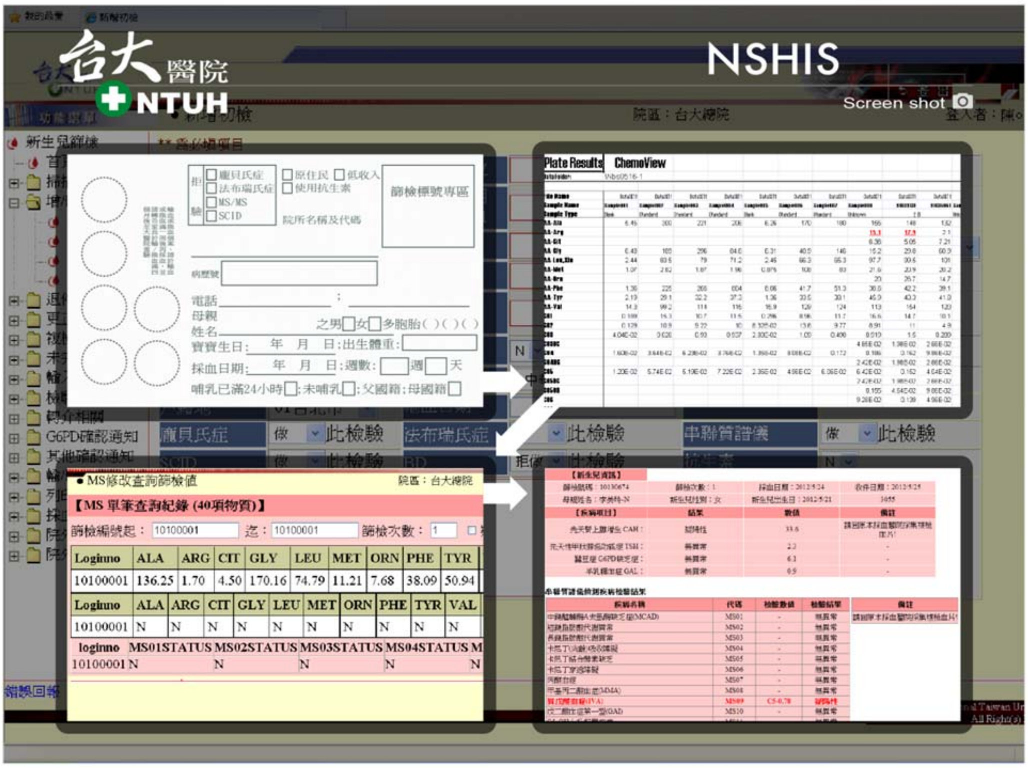

Figure 1. Snapshot of newborn screening hospital information system

In **Figure 1**, the snapshots illustrate the screening daily operations at NTUH Newborn Screening Center after received specimens via post mails. At this point, the initial sample registration has been completed. The yellow selections at left of the figure, in localized language, i.e. traditional Chinese, provide the list of Subsystems' functionalities. For example, after selecting the "barcode scanning" button and scanning a newborn's filter paper, the newborn's demographic data with sampling information pop up as indicated at the upper left corner. The neonate specimens under taken MS/MS process, selecting the Sample Screening Subsystem functions, the concentration compound raw data, i.e., an Excel file, is created as shown at the upper right corner of the figure. After uploading the Excel file as inputs into the system, the screening outcomes are generated and displayed at lower left corner of the diagram. From the outcomes, the screening reports are presented as indicated at lower right corner of the diagram. The reports can be retrieved and reviewed by neonatal parents.

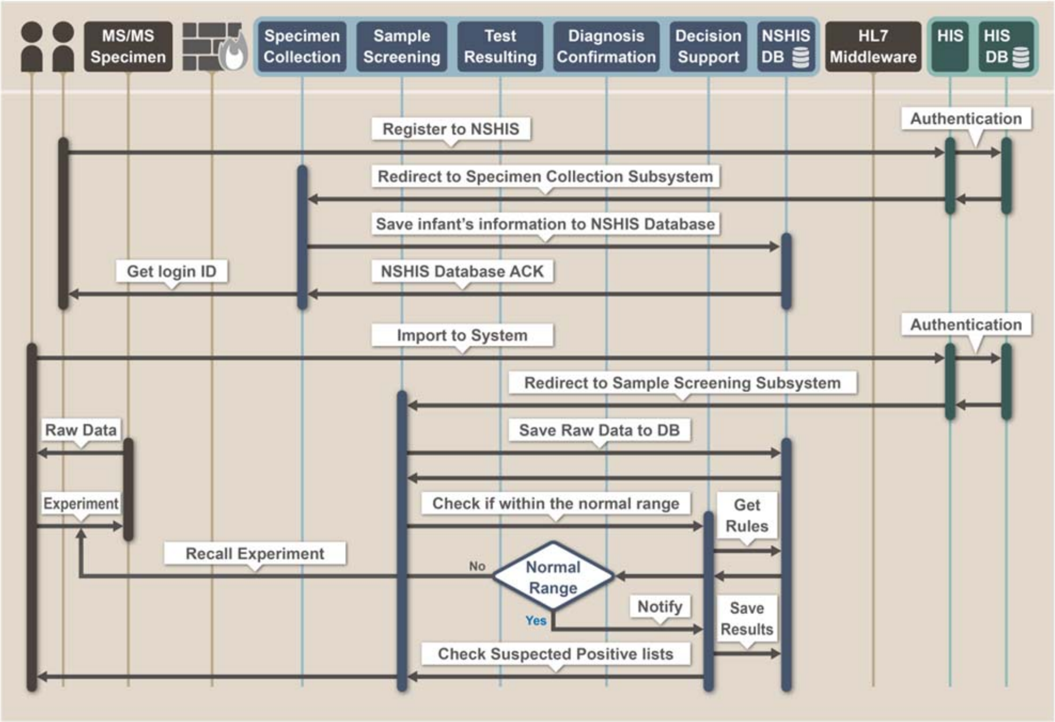

Figure 2. The collaborating, interoperability among NSHIS subsystems

In **Figure 2**, the sequence presents the collaborating, interoperability among NSHIS subsystems. Users of phlebotomy clinics login the system by authentication/authorization via web services checking the personal (security) data stored previously in the database of NTUH HIS. After login, the web page will be redirected to the NSHIS portal in order to handle the initial Specimen Collection steps. In addition, the infant's demographic data are stored into NSHIS database, and a barcode is generated. Meanwhile, the experimental raw data generated by the MS/MS will be uploaded to the NSHIS database as well as performing Sample Screening processes to execute the data analysis. The Diagnosis and Confirmation subsystem adjusts whether the raw data are in normal ranges, determines redoing experiments if necessary. Moreover, the Data Mining and Decision Support subsystem can be used to refine the screening statistics, accuracy as well as quality controls.
